# The Mechanism of Pain in Migraine

**Published:** 1905-07-01

**Authors:** 


### HOSPITAL CLINICS.

### THE MECHANISM OF PAIN IN MIGRAINE.

THE mechanism of pain in this disease, which is as much as to say its pathology, has remained obscure in spite of the wealth of imagination which has been lavished upon it. Liveing, for instance, held that migraine was a nerve-storm and the sensory equivalent of epilepsy, and there is no doubt that the two not infrequently concur in the same individual, or attack different members of the same family, while both are equally prone to begin in early youth. On the other hand there is the important distinction that migraine tends towards spontaneous cessation as age advances, while epilepsy, if it endures to puberty, is seldom entirely in abeyance. A majority of authors, however, incline to a vaso-motor explanation for the disease; thus Du Bois-Raymond considered migraine to signify a tetanus of the sympathetic, that is to say, a local vaso-dilation; Möllendorf favoured a vaso-constriction due to paralysis of the sympathetic in the neck, while Brunton, influenced by his own case, argues for a vaso-constriction, but a localised as opposed to Möllendorf's generalised one.

Dr. Francis Hare [1] joins the roll of believers in vaso-dilation. He considers migraine to be the true analogue of dyspnœa in spasmodic asthma, *i.e.* a condition depending proximately upon vascular distension at the seat of pain, this distension being compensatory of, and exaggerated by, a wide-spread peripheral vaso-constriction. In support of this thesis he cites certain descriptions of the attack; thus Anstie says that the feet are actually as well as subjectively cold; Möllendorf speaks of the "icy coldness of the hands and feet, and the shivering of the surface generally"; Dr. Hare considers it conclusively proved that vaso-constriction is not the proximate cause of the pain in migraine, for though the extremities be markedly cold during an attack, they are never painful. Arguing for vaso-dilation, he points out that there is no more certain means of causing vascular distension at any point than by a local dilation associated with a general constriction. By this means not only is the blood sent to the dilated area in unnatural abundance, but it is delivered with the additional force supplied by a heightened blood-pressure. Now wherever swelling can freely take place vascular distension is accompanied by little or no pain. This is a clinical commonplace, well illustrated by the difference in the degree of pain that marks a furuncle on the loose skin of the back as opposed to a similar lesion in the dense tissues of the external auditory meatus. This being so, it is easy to see that anything causing vascular distension in such unyielding tissues as the dura mater, pericranium, or scalp, selects a site where the resulting pain cannot fail to be of a high degree of severity.

Vascular distension, then, of more or less localised areas of the cranial vault is to be considered, according to this view, as the proximate cause of the pain in migraine, and the author supports his thesis by the following clinical observations. Pressure on the arterial trunks supplying the seat of pain causes immediate relief. "If the common carotid on the affected side be compressed at the level of the thyroid cartilage the headache vanishes as if by magic," says Möllendorf, and Dr. Hare fully bears out the observation. The latter asserts that in unilateral migraine pressure upon the common carotid on the same side immediately and completely removes the pain, which reappears as soon as the pressure is relaxed; that in bilateral attacks pressure on one artery discharges the pain completely from that side and increases it on the opposite side; that in bilateral occipital migraine cessation of the pain may be brought about by pressure upon both occipital arteries, while it is made unilateral by pressure on one of them; and, finally, that pancranial migraine may be entirely suppressed for the time by pressure exerted upon both occipital and both superficial temporal arteries.

These interesting statements are corroborated by certain further observations upon migrainous subjects: thus the well-known alleviation of the headache which follows immediately upon the act of vomiting, would receive an explanation on the ground that this act depresses the circulation to such an extent that the distended area has immediate relief; again, it is a common practice for migrainous individuals to apply cold compresses to the affected side of the head during the attack, knowing from experience that such treatment results in an abatement of the pain; pressure upon the affected region is also commonly resorted to spontaneously by the sufferers from this malady, and the author mentions the case of one lady who was so much impressed by the relief to be thus obtained that she projected a clip which would automatically exert the necessary pressure. This last observation tallies very closely with the common experience in regard to alveolar abscess, namely, that while the neighbouring tooth is very tender to slight pressure, yet firm pressure upon it, as by clenching the teeth, gives a very definite though transient comfort. Lastly, Sir Lauder Brunton holds that a considerable number of migrainous people are eased by the administration of general vaso-dilators, the chief of which is nitro-glycerine. This, however, is not always so, as we can personally testify. We may mention in conclusion a very interesting property occasionally manifested by migraine—namely, the capacity for appearing metastatically. Thus, a patient of Valleix who was in the habit of seeking relief in cold compresses, found on one occasion that the pain disappeared entirely from the head, but was shortly followed by an epigastric uneasiness, which presently developed into most agonising pain associated with a sense of suffocation and ineffectual attempts to vomit—a state of affairs which finds a curious parallel in the occasional behaviour of gout.

[1] Med. Press and Circular, June 7, 1905.

